# Cancer Cachexia: Mechanisms and Clinical Implications

**DOI:** 10.1155/2011/601434

**Published:** 2011-06-13

**Authors:** Claire L. Donohoe, Aoife M. Ryan, John V. Reynolds

**Affiliations:** ^1^Department of Surgery, Trinity Centre for Health Sciences, Trinity College Dublin, St James' Hospital, Dublin 8, Ireland; ^2^Department of Nutrition, Food Studies and Public Health, New York University, New York, NY 10003, USA

## Abstract

Cachexia is a multifactorial process of skeletal muscle and adipose tissue atrophy resulting in progressive weight loss. It is associated with poor quality of life, poor physical function, and poor prognosis in cancer patients. It involves multiple pathways: procachectic and proinflammatory signals from tumour cells, systemic inflammation in the host, and widespread metabolic changes (increased resting energy expenditure and alterations in metabolism of protein, fat, and carbohydrate). Whether it is primarily driven by the tumour or as a result of the host response to the tumour has yet to be fully elucidated. Cachexia is compounded by anorexia and the relationship between these two entities has not been clarified fully. Inconsistencies in the definition of cachexia have limited the epidemiological characterisation of the condition and there has been slow progress in identifying therapeutic agents and trialling them in the clinical setting. Understanding the complex interplay of tumour and host factors will uncover new therapeutic targets.

## 1. Introduction

The etymology of the word cachexia points to its association with poor prognosis: it is derived from the Greek *kakos* and *hexia*—“bad condition” and has long been recognised as a key sign in many cancers. It is a multifactorial condition which comprises skeletal muscle and adipose tissue loss which may be compounded by anorexia, a dysregulated metabolic state with increased basal energy expenditure and is resistant to conventional nutritional support. The pathophysiological mechanisms have begun to be elucidated and this has led to developments in therapeutic avenues [1]. 

Cachexia correlates with poor performance status, poor quality of life, and a high mortality rate in cancer patients [2]. In a meta-analysis of studies pertaining to patients with advanced cancer and survival of less than 90 days, symptoms including weight loss and anorexia correlated with poor prognosis [3]. Loss of greater than 5–10% of body weight is usually taken as a defining point for cachexia, although the physiological changes may be present long before this cutoff point is reached. Furthermore, the degree of weight loss which significantly impacts on prognosis or performance has not been defined. A longitudinal study has shown that 2.5 kg weight change over 6–8 weeks is sufficient to produce significant changes in performance status [4]. Death usually occurs when there is 30% weight loss [5]. 

The prominent clinical feature of cachexia is weight loss in adults (corrected for fluid retention) or growth failure in children (excluding endocrine disorders). Anorexia, inflammation, insulin resistance, and increased muscle protein breakdown are frequently associated with cachexia [6]. However, there is no clear consensus definition of this common problem in cancer patients leading to a poor understanding of the aetiology of the condition. Earlier definitions of cachexia described “a wasting syndrome involving loss of muscle and fat directly caused by tumour factors, or indirectly caused by an aberrant host response to tumour presence” [7], however more recent definitions have downplayed the importance of fat loss and describe cachexia as “a complex metabolic syndrome associated with underlying illness and characterised by loss of muscle with or without loss of fat mass” [6], thus highlighting the unique consequences of muscle wasting—the hallmark of cachexia. Without an established definition, future studies in this area will be hampered. A recent consensus definition has been proposed to include further factors to diagnose the cachexia syndrome such as involuntary weight loss, decreased muscle mass, anorexia, and biochemical alterations (*C*-Reactive Protein (CRP), albumin, haemoglobin [8]).

One such study looked at 170 pancreatic cancer patients with weight loss >5% and whether a triad of >10% weight loss, low food intake (<1500 kcal/day), and systemic inflammation (CRP > 10 mg/dL) could better predict adverse functional outcome as well as poor prognosis versus weight loss alone [8]. When two of three of these criteria were present, (representing 60% of the patients) a cohort of patients with adverse function and prognosis were identified [8]. 

The prevalence of cachexia is thought to be up to 80% of upper gastrointestinal cancer patients and 60% of lung cancer patients at the time of diagnosis [9]. There are no clear figures for the estimated prevalence within specific cancer cohorts. When the electronic medical records of over 8500 patients with a wide variety of malignancies were analysed for the prevalence of cachexia amongst the cohort, the proportion varied according to which standard definition was used: 2.4% using the World Health Organisation's International Classification of Diseases (ICD) cachexia diagnostic code; 5.5% for the ICD diagnosis of cachexia, anorexia, abnormal weight, and feeding difficulties; 6.4% were prescribed megestrol acetate, oxandrolone, somatropin, or dronabinol; 14.7% had >5% weight loss [10]. Despite methodological flaws, there was an interesting lack of overlap between the different criteria pointing to the underdiagnosis of cachexia in clinical practice.

Decreased muscle strength may help distinguish cachexia from other causes of anorexia and fatigue in cancer patients [11]. Decreased muscle strength could be used as a diagnostic criterion with greater sensitivity and specificity for cancer cachexia. Cancer patients who are losing weight and have a systemic inflammatory response have poorer performance status [4]. Until a clear definition with well-defined cut-offs emerges, identification and treatment of cachectic patients as well as research in the area will remain limited. A new consensus definition for diagnostic purposes has been suggested and is outlined in Table 1 [6].

## 2. Pathophysiology

**Figure 1:**
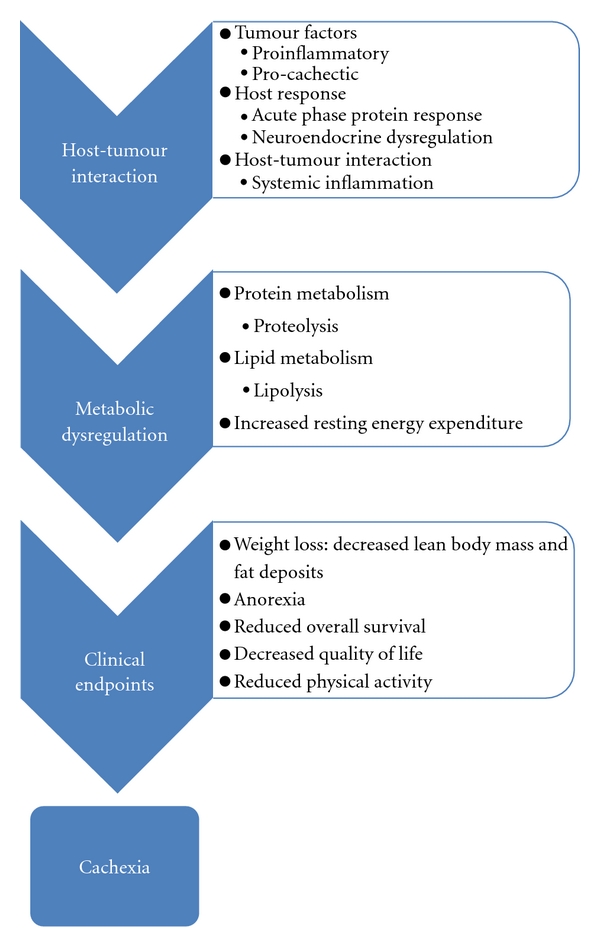
Clinical consequences of cancer cachexia.

Pathophysiological changes and clinical consequences of cachexia are summarised in Figure 1.

### 2.1. Metabolic Changes

The metabolic changes found in cachexia resemble those of infection rather than starvation [12] and are multifactorial and complex. Weight loss of cancer cachexia is due to loss of both skeletal muscle and adipose tissue mass, whereas weight loss is mainly from adipose tissue stores in starvation [13]. In cachexia there is an increase in muscle protein catabolism leading to net loss of muscle mass. The ATP ubiquitin-dependent proteolytic pathway is the greatest contributor to proteolysis in cachexia [14, 15]. Other proteolytic pathways such as lysosomal cathepsins B, H, D, and L [16] and activity of the calcium/calpain pathway have also been implicated [17]. Increased intracellular proteolytic activity usually manifests as loss of body weight. This proteolysis has been shown to occur even in the absence of weight loss in cancer patients. Activation of proteolysis is an early event during tumour growth and it may be present for a long time prior to its clinical manifestation. Protein synthesis may be increased or unchanged [18]. 

Loss of adipose tissue mass is due to lipolysis [5]. This process is driven by lipid mobilising factor (LMF) and tumour (and host) factor zinc-alpha-2 glycoprotein which has a direct lipolytic effect and sensitises adipocytes to lipolytic stimuli and shows increased expression in cachexia [19]. A further compounding factor is the increased resting energy expenditure due to the dysregulation of energy metabolism. Cancer patients have a higher resting energy expenditure than noncancer controls [20]. It has been speculated that this is due to altered gene expression of mitochondrial membrane uncoupling proteins which uncouple respiration from ATP production resulting in loss of energy as heat [5].

The metabolic changes seen in cachexia are a result of the interplay of tumour factors, host factors, and the interaction between the two.

### 2.2. Tumour Factors

Tumour cells produce both pro-inflammatory and procachectic factors, which stimulate a host inflammatory response [1]. Tumour produced procachectic factors include proteolysis-inducing [45] and Lipid-mobilising factors [46]. PIF has been identified in the urine of weight losing patients with pancreatic, colon, lung, ovarian, breast, and liver cancers [47]. In animals, PIF signals via NF*κ*B and STAT3 pathways [48]. Stimulation of these pathways, induces proteolysis in muscles via the ubiquitin-proteasome pathway [49] and in hepatocytes, results in production of IL-6, IL-8 and CRP [48]. Tumour xenografts expressing human PIF do not induce cachexia in mice [50]. Further attempts to correlate PIF levels and outcomes have not shown any correlation [51]. Therefore the proposed mechanisms of PIF have not yet been validated in humans. Parathyroid hormone-related peptide (PTHrP), another tumour-derived circulating factor, is associated with higher soluble tumour necrosis factor receptor levels and with lower albumin and transferrin levels [52].

Lipid mobilising factor has been found in cancer patients losing weight but not in those with stable weight [53]. It is thought that LMF sensitises adipocytes to lipolytic stimuli by increasing cyclic AMP production [54]. LMF may bind to beta adrenergic receptors and causes either increased receptor number or increased G protein expression [55]. 

### 2.3. Host-Tumour Interaction

Inflammatory cytokine production by the tumour microenvironment in response to tumour cells may drive the cachexia process. Rodent tumour models display increased systemic inflammatory cytokine production, which correlates with the amount of weight loss [56, 57]. The murine model of cancer cachexia associated with systemic inflammation suggests that there is an interplay between IL-1 *β* and IL-6 within the tumour microenvironment, which leads to their amplification [58]. Reduction of IFN-*γ* by monoclonal antibody treatment reverses cachexia in the Lewis lung carcinoma in mice [59].

Pro-inflammatory cytokines produced include TNF-*α*, IL-1 and IL-6 [1]. It is not certain whether the cytokine production is primarily from tumour or host inflammatory cells. It has been hypothesised that either tumour cell production of pro-inflammatory cytokines or the host inflammatory cell response to tumour cells is the source of the acute phase protein response seen in many malignancies and in cachexia. One study of oesophagogastric cancers showed cytokine protein concentrations of IL-1*β*, IL-6 and TNF-*α* are significantly elevated in tumour tissue. Tumour tissue concentrations of IL-1*β* protein correlated with serum CRP concentrations (*r* = 0.31, *P* = .05; linear regression) and tumours with diffuse or patchy inflammatory cellular infiltrate were associated with elevated serum CRP [60]. Similarly the production of IL-6 by Peripheral Blood Mononuclear Cells (PBMCs) in pancreatic cancer patients induced an acute phase protein response in another study [61]. Martignoni et al. have suggested that IL-6-overexpression in cachectic pancreatic cancer patients is related to the ability of IL-6 producing tumours to sensitise PBMC and induce IL-6 expression in PBMCs [62].

TNF-alpha and the tumour factor proteolysis-inducing factor are the major contenders for skeletal muscle atrophy in cachectic patient. They both increase protein degradation through the ubiquitin-proteasome pathway and depress protein synthesis through phosphorylation of eukaryotic initiation factor 2 alpha [19]. Studies have shown that proteolysis-inducing factor levels correlate with the appearance of cachexia, but there is some disagreement regarding a correlation between serum levels of TNF-alpha and weight loss. Furthermore, only antagonists to proteolysis-inducing factor prevent muscle loss in cancer patients, suggesting that tumour factors are the most important.

### 2.4. Host Response Factors

#### 2.4.1. Acute Phase Protein Response

Systemic changes in response to inflammation are denoted the acute phase response [63]. Up to 50% of patients with solid epithelial cancers may have an elevated acute phase protein response [64]. This acute phase protein response (APPR) has been associated with hypermetabolism: in pancreatic cancer patients APPR correlated with elevated resting energy expenditure and reduced energy intake [65]. Other longitudinal studies have found a poorer prognosis in patients displaying this response, independent of weight loss [66]. *C*-reactive protein (CRP) is the most prevalent method used to assess the magnitude of the systemic inflammatory response [63]. The modified Glasgow prognostic score (mGPS) (Table 2) combines CRP and albumin concentrations to create a simple scoring system which is a prognostic factor independent of stage and treatment and predicts survival [21, 67].

Raised CRP concentrations at the time of admission to hospital are indicative of an increased risk for all-cause mortality; there is a 22.8-fold increase in cancer mortality in patients with highly elevated CRP concentrations (>80 mg/L) [68]. This response appears to be prevalent amongst cancer patients with elevated CRP measured in almost 80% of 106 patients with inoperable nonsmall cell lung cancer (NSCLC), 40% of whom had >5% weight loss [69]. In patients without weight loss, those who displayed evidence of a systemic inflammatory response reported more fatigue (*P* < .05) [69]. In patients with gastro-oesophageal cancer, the rate of weight loss correlates with serum concentrations of *C*-reactive protein [70]. Elevated CRP levels at the time of diagnosis has been found to be a predictor of poor prognosis in pancreatic, lung, melanoma, multiple myeloma, lymphoma, ovarian, renal, and gastrointestinal tumours [71].

The exact mechanisms linking cachexia, APPR, and poor outcomes is not known. It may be that this systemic alteration in protein metabolism drives the proteolysis of skeletal muscle to fuel the switch to acute phase reactant production. The APPR requires large amounts of essential amino acids: 2.6 g of muscle protein must be catabolised to produce 1 g of fibrinogen [72].

#### 2.4.2. Neuroendocrine Factors

A number of neuroendocrine factors appear to be dysregulated in the cancer state resulting in insulin resistance, reduced anabolic activity, and elevated cortisol [47]. This dysregulation may be driven by the systemic inflammatory response associated with cancer. Inflammatory cytokines such as TNF-*α* and IL-6 have been implicated in insulin resistance [73]. The endogenous production of or response to anabolic growth factors in patients may be affected either by the tumour or the host response to the tumour and may contribute to cachexia. Testosterone or derivatives have been shown to increase protein synthesis and muscle mass [74]. Emerging evidence implicates reduction in insulin-like growth factor 1 in cachectic states [75].

### 2.5. Anorexia and Cachexia: An Interdependent Relationship?

Whilst loss of appetite and resultant decrease in energy intake undoubtedly contribute to weight loss associated with cancer cachexia, whether anorexia occurs by an independent process or is a result of the inflammatory process of cachexia is not fully understood. Anorexia itself may have a number of components—nausea, altered taste sensation, swallowing difficulties, or depression. The failure of aggressive supplementary nutritional regimes to reverse weight loss in many patients points to primacy of the cachexia disease process [5] and in fact, this disease process may act to establish anorexia. It is thought that lack of appetite is secondary to factors produced by the tumour or the immune response to the tumour. Specifically, cytokines may inhibit the neuropeptide *Y* pathway or mimic negative feedback action of leptin on the hypothalamus, leading to anorexia [76, 77].

In a study of patients with gastro-oesophageal malignancy (*n* = 220), 83% of whom had weight loss, multiple regression identified dietary intake (estimate of effect: 38%), serum CRP concentration (estimate of effect: 34%), and stage of disease (estimate of effect: 28%) as independent variables in weight loss in these patients [70]. If serum CRP is taken as a proxy measure of systemic inflammation due to cancer cachexia, this indicates that weight loss in cancer is not merely due to reduced calorie intake. 

Recently, understanding of the physiological mechanisms of appetite regulation has been increasing. There are two sets of neurons within the arcuate nucleus of the hypothalamus identified to be involved: the melanocortin system and the neuropeptide Y system. Neuropeptide Y stimulates appetite on its own or via release of other orexigenic proteins [78]. Neurons which release *α*-melanocyte-stimulating hormone (*α*-MSH) and signal via melanocortin-3 and 4 receptors (MC3R, MC4R) result in decrease in food-seeking behaviour, increased basal metabolic rate and decreased lean body mass [79, 80]. These neurons are constitutively active as mutation in the MC4R results in childhood obesity [81]. Agouti-related protein (AgRP) is produced by neurons (which also produce neuropeptide Y) and counteracts the action of MC4R-stimulating proteins promoting appetite [82]. These “appetite neurons” also express receptors for circulating leptin [83] and interleukin-1*β* (IL-1*β*) [84], both of which downregulate appetite and receptors for ghrelin (the orexigenic protein, which increases AgRP) [85].

## 3. Consequences

Cachexia results in a state of active inflammation whereby tumour-derived factors and the aberrant host response to these factors result in a catabolic state. Whether this catabolic state is the ultimate cause of death in some patients is unknown although a substantial proportion of cancer patients die with symptoms of advanced cachexia [9]. Cachexia directly impacts overall survival, quality of life, and physical activity. 

### 3.1. Survival

Weight loss has been indicated as an important prognostic factor for cancer patients. A classic study by DeWys and colleagues underscores the impact and outcome of weight loss in cancer patients [2]. Using retrospective evaluation in a multicentre study of more than 3000 patients with different tumour types, these researchers reported moderate to severe weight loss in 30% to 70% of patients, depending on the tumor type. The amount of weight loss depends upon tumor site, size, type, and stage. Age and treatment type also play a role. The greatest incidence of weight loss was seen among patients with solid tumours, for example, gastric, pancreatic, lung, colorectal, and head and neck. Patients with solid tumours are often likely to lose 10% or more of their usual body weight. There is a lower risk of weight loss in patients with breast and hematological cancers. Within each tumour type, survival times were shorter for patients who had experienced weight loss than in those who did not. Not only did weight loss predict overall survival, but it also indicated a trend towards lower chemotherapy response rates.

In more recent studies, similar findings of reduced survival have been reported. Buccheri and Ferrigno (2001) [86] reported in 388 NSCLC cases that total weight loss was the best indicator of prognosis. In ovarian cancer Hess et al. (2007) [87] found a significant relationship between weight change and survival—on multivariate analysis the risk of death increased by 7% for each 5% drop of body weight. In Gastro-oesophageal cancer Deans and Wigmore (2009) [71] reported that patients with the lowest rate of weight loss had a median survival of 30.2 months versus 7.5 months in those with the highest rate of weight loss. Similar findings have also been reported in pancreatic cancer [88]. 

One proposed mechanism to explain why patients with weight loss have a poorer survival is the increased incidence of complications from surgical, radiotherapeutic, and chemotherapeutic treatments. In a study by Andreyev et al. [89], 1555 patients with a number of different gastrointestinal tumour types were analysed to examine whether weight loss affected prognosis. In patients with weight loss: chemotherapy doses were lower; they developed more frequent and more severe dose limiting toxicity and received, on average, one month less chemotherapy (*P* < .001 in all). Weight loss correlated with shorter failure-free survival, overall survival, decreased response, quality of life, and performance status (*P* < .001 in all) [89]. Whether reduced survival is due to a more aggressive tumour profile in patients with weight loss or due to suboptimal treatment related to weight loss, remains unknown.

### 3.2. Quality of Life

Cachexia contributes substantially to morbidity in cancer patients. It is associated with symptoms such as fatigue, weakness, poor physical performance, and thus leads to a lower self-rated quality of life. Indeed, when the impact of various factors is related to self-rated quality of life scores, the proportion determined by weight loss is 30% and by nutritional intake 20%, compared to cancer location (30%), disease duration (3%), and stage (1%) [90]. Patients who continue to lose weight while receiving palliative chemotherapy have reduced global quality of life and performance scores when compared to those whose weight loss stabilises [91].

### 3.3. Physical Activity

Physical activity has been described as a novel, objective, and robust functional outcome measure that is frequently impaired in cachectic states [92]. Activity levels are influenced by several conventional quality of life domains. Measurement of physical activity has long represented a challenge for researchers using time-consuming and expensive tools such as doubly labelled water and indirect calorimetry. However research using these methods has revealed that although resting energy expenditure may be elevated in cachectic patients, total energy expenditure is reduced because weight-losing cancer patients reduce the magnitude of their energy deficit through reductions in physical activity. This reduction in physical activity can be significant—in one study the measured mean physical activity rate was equivalent to that of spinal cord injury patients living at home and greatly reduced versus normal controls [93]. In a more recent study by Dahele et al. (2007) [94] using advanced ambulatory pedometer technology, cancer patients receiving palliative chemotherapy were shown to spend significantly more time lying and sitting, and significantly less time in quiet standing or stepping compared with controls, taking on average 43% less steps than healthy controls. It is known that bed rest leads to a decrease in skeletal muscle mass in healthy patients, due to reduced protein synthesis [95]. Thus, loss of physical function results in decreases in performance status, ability to perform activities of daily living, decreased social interactions, and alterations in body image, all of which manifest as reduced quality of life [96]. Interventions which increase physical activity would be anticipated to be highly beneficial.

Antineoplastic therapies such as surgery, radiotherapy and chemotherapy, may also impact on the development of systemic inflammation and particularly may impact on swallowing difficulties and anorexia due to nausea [97].

## 4. Therapeutic Approaches

### 4.1. Goals of Therapy

Clearly since cancer cachexia is associated with a poor prognosis, the aim of management is often to improve symptoms and quality of life. It is noted that a response to chemotherapeutic treatment by shrinkage of the tumour burden often leads to improvement in the cachectic state. The primary endpoints of optimal treatment of cancer cachexia are improvements in lean body mass, resting energy expenditure, fatigue, anorexia, quality of life, performance status, and a reduction in pro-inflammatory cytokines.

A greater understanding of the process of inflammation and its fundamental role in the development of cachexia has led to new avenues opening up in the approach to management of the condition. The hypothesis is that effective treatment of cancer cachexia will improve performance status and quality of life and by inhibiting the process driving cachexia, survival may be improved. In patients who stop losing weight while receiving chemotherapy for gastrointestinal cancers, median survival is improved (15.7 months versus 8.1 months, *P* = .0004) [89]. Animal models are generally unsatisfactory models for assessing the efficacy of intervention due to the larger proportional size and the aggressive doubling rate of tumours: thus the biological behaviour is different to that seen in the clinical setting [98]. 

There has been recent progress in producing trials of high clinical quality for licensing purposes but these trials may be beset by difficulties in adequate endpoint analysis due to the numbers lost to followup or patients being unable to comply with therapy due to their poor overall condition, thus limiting their duration, power, or generalisability [99, 100]. In addition there is a degree of heterogeneity in defining relevant end points for analysis of intervention in cancer cachexia.  Table 3 summarises the range of endpoints which may be used. One study of 388 nonsmall cell lung cancer patients found that total weight loss was the best predictor of prognosis rather than speed of weight loss [101]. However, weight loss alone does not identify the full effect of cachexia on physical function [8]. It is the loss of fat-free mass (FFM) that is responsible for the reduced functional status, increased mortality, and other negative outcomes associated with malnutrition [102]. Body fat is easier to gain than FFM, so studies that show improved body weight may not translate into reductions in morbidity or improvements in functional status. To improve functional ability and hence quality of life patients need not only to become weight stable but regain the lean tissue lost in the cachectic process. Thus, interventions which lead to improvements in functional status would be expected to cause increases in lean body mass rather than fat mass, however, this distinction is often not reported in interventions. 

The strong impact that cancer cachexia has on cancer patients' outcome and quality of life suggests that nutritional issues should be taken into consideration from the beginning of the natural history of cancer, a concept termed the parallel pathway [103]. Indeed studies of nutritional intervention that have reported a better weight maintenance in patients are in those who are treated in the “precachexia” phase, that is, prior to loss of >10% of body weight and prior to elevations of CRP. Dietary counselling with or without oral nutritional supplements has proven efficacy in stabilising nutritional status in pre-cachectic patients [104, 105]. A nutritional assessment to seek reversible causes of weight loss is the first step in management in cachectic patients. Approximately 40% of cancer patients eat less than the 34 kcal/kg/day required to maintain weight [106]. The European Society of Parenteral and Enteral Nutrition (ESPEN) report in a consensus statement that there is Grade A evidence for intensive dietary counselling with food plus or minus oral nutritional supplements in preventing therapy-associated weight loss, preventing treatment interruptions and increasing dietary intake in gastrointestinal or head and neck cancer patients undergoing radio- or chemotherapy [107]. 

For patients with advanced cachexia (>10% weight loss, systemic inflammation and poor appetite) studies seeking to assess the effect of targeted nutritional advice and supplements have generally reported no significant improvement in nutritional status. Standard enteral or parenteral supplements do not appear to result in lean mass weight gain for the typical cancer patient [5, 98, 108]. The largest evaluation of the literature regarding nutritional supplementation (NS) (oral or tube) in cancer patients was the systematic review by Elia et al. (2006) showing no difference in mortality in patients undergoing chemotherapy/radiotherapy (4 RCTs) or surgery (4 RCTs) [109]. A systematic review of parenteral nutrition in cancer patients showed no difference in mortality (19 RCTs), increase in total complication rates in those given parenteral nutrition (8 RCTs), and significantly lower tumour response rate in patients receiving parenteral nutrition (15 RCTs) [110]. 

This is likely because the inflammatory response of cachexia prevents anabolism. In many cases an attempt is being made to reverse or halt a rapidly advancing catabolic process and it is unrealistic to expect a reversal with calories and protein alone. 

The poor results observed with conventional nutrition support in cachectic patients led to the emergence of so-called nutraceuticals or immunonutrition supplements, in an attempt to nutritionally modify the metabolic milieu by providing anti-inflammatory substances, such as eicosapentaenoic acid (EPA), at levels much higher than that typically found in the diet. 

### 4.2. Eicosapentaenoic Acid

Eicosapentaenoic acid (EPA), a long-chain polyunsaturated fatty acid (PUFA) of the omega-3 (n-3) family, has been studies in relation to cancer cachexia for over 15 years. It is of interest in the context of cancer cachexia as it has potential to impact on both the underlying metabolic abnormalities of tumour-induced weight loss, as well as modulation of immune function. When EPA is consumed at levels above that normally found in the diet, it replaces arachidonic acid (AA), an n-6 PUFA, in cell membrane phospholipids. It then acts as a substrate for the production of the 3 series prostaglandins and the 5 series leukotrienes. Eicosanoids synthesized from the n-3 PUFAs (i.e., EPA) rather than the n-6 PUFAs (i.e., AA) have lower potential for promoting inflammation. Modulation of dietary fatty acids can therefore have an impact on many immune processes such as proliferation, phagocytosis, cytotoxicity, and cytokine production [111].

Despite initial studies showing anabolic effects, principally gains of lean body mass, improvements in grip strength, quality of life, and reductions in IL-6 and PIF could be achieved in a variety of cancers [99], including pancreatic cancer [112, 113], lung cancer [114], and colorectal cancer [115], analysis of RCTs only, using the Cochrane approach, did not show any differences between EPA supplementation and placebo [39]. Whether this is a true representation or a reflection of the advanced cachexia of participants or inherent differences in EPA metabolism between individuals (with only a proportion of patients able to respond to EPA) needs further examination. On subgroup analysis, patients who comply with EPA supplementation seem to have improved lean body mass [116]. 

EPA-enriched oral nutritional supplements (ONSs) have been compared to megestrol acetate in the North Central Cancer Treatment Group trial of 421 patients with weight loss, poor intake, and anorexia [117]. In a 3-month intervention period, patients were randomized to either EPA-enriched ONS plus placebo liquid suspension, standard ONS plus megestrol acetate suspension, or EPA-enriched ONS plus megestrol acetate suspension. Weight gain was highest in the megestrol acetate group but unfortunately body composition was not assessed and so changes in water weight cannot be controlled for. There was no difference in survival, appetite, or quality of life scores between the groups, however patients on megestrol acetate reported higher rates of impotence. The fact that an EPA enriched ONS scored as well as drug therapy on certain clinical endpoints (e.g., survival and global quality of life) underscores the limitations of each treatment. 

*β*-hydroxyl *β*-methyl butyrate (HMB), glutamine, and arginine supplementation have been combined in the hope of a synergistic effect of HMB (a modulator of protein turnover) and the amino acids (immunomodulatory) would increase weight. A phase III RCT of this combination did not show any difference in lean body mass between control and intervention groups [100]. 

### 4.3. Pharmacological Agents

Pharmacological options are summarised in Table 4. Among orexigenic agents, megestrol acetate is by far the most widely prescribed and at least 15 randomised controlled clinical trials have demonstrated that this drug, at doses ranging from 160–1600 mg/d significantly improves appetite with respect to placebo [118]. A recent Cochrane meta-analysis reported that it improves weight gain and appetite in cancer patients [29]. Although this increase in appetite is very desirable for both patients and their carers, in most of these trials no definitive improvement in global quality of life was observed [29]. 

Anti-inflammatory agents (COX inhibitors) can reduce weight loss and aid maintenance of performance status in advanced cancer [119]. The COX-2 inhibitor, meloxicam showed activity against PIF-induced proteolysis, prior to its withdrawal from the market [120]. Beta-adrenoreceptor blockade can reduce resting energy expenditure in patients with cancer (*n* = 10) but have not been trialled in larger-scale studies [121]. They are thought to inhibit proteolysis and lipolysis [122] and have been shown to downregulate catecholamine-induced catabolism in burns patients [123]. Agents which reduced cytokine levels such as thalidomide and pentoxifylline have only shown modest or minimal activity. At RCT, thalidomide has been shown to attenuate weight loss and lead to improved physical function [35]. Pentoxifylline did not have any clinical benefit. Specific antitumour necrosis factor- (TNF-)*α* agents, etanercept and infliximab, did not show any positive effect on appetite or body weight in RCTs [124, 125]. Corticosteroids, although widely used, have significant side effects including protein breakdown, insulin resistance, water retention, and adrenal suppression and tend to be used during the preterminal phase of patient illness [23, 126]. Anabolic steroid derivatives such as nandrolone and oxandrolone have not been studied in clinical trials in a cancer cohort. Insulin [27], ATP infusions [28], and melatonin [41] have produced modest positive effects in small clinical trials and require further substantiation.

### 4.4. Combination Therapy

In unresectable cancer cases, there is currently no goal standard treatment that can attenuate catabolism and inflammation, stimulate appetite and intake and consequently promote anabolism (specifically of lean body mass). A multimodal approach has therefore been advocated in the treatment of cancer cachexia. Mantovani (2010) randomised 332 patients with cancer-related anorexia/cachexia syndrome to one of five arms of treatment: (1) medroxyprogesterone 500 mg/d or megestrol acetate 320 mg/d; (2) oral supplementation with eicosapentaenoic acid (EPA); (3) L-carnitine 4 g/d; (4) thalidomide 200 mg/d; (5) a combination of the above for a total of 4 months [127]. Results showed the superiority of arm 5 over the others for all primary endpoints. Significant improvements were observed in arm 5 in LBM, fatigue scores, appetite, and total energy and active energy expenditure with REE decreasing significantly. Toxicity was negligible and comparable between treatment arms.

### 4.5. Potential Therapeutic Targets

Due to the lack of clinical efficacy of agents which seemed promising in the laboratory setting, ongoing research has continued to explore new therapeutic targets and to develop new agents. Much of this has focussed on manipulation of the melanocortin system of appetite regulation [128]. Activation of the Melanocortin-4-receptor (MC4R) in murine models decreases food-seeking behaviour, increases basal metabolic rate, and decreases lean body mass [80]. Treatment with a MC4R antagonist attenuated these responses [79]. Ghrelin induces the release of growth hormone, regulates appetite, and has anti-inflammatory properties [129, 130]. Initial human studies in Phase I open trials have confirmed safety and show some increase in appetite and body weight [131]. Myostatin is a growth factor involved in the normal regulation of muscle mass [132]. Myostatin inhibitors and IL-6 antagonists are currently at Phase I RCT stage in development [131].

## 5. Conclusions

A consensus definition incorporating clinical, functional, and biochemical parameters is necessary in order to adequately identify and treat patients with cancer cachexia. A greater understanding of the pathophysiology, particularly in terms of the processes which drive cachexia will lead to new therapeutic target development. A number of issues remain to be resolved including whether inflammation drives the process or is a byproduct of the process. Does reversal of weight loss alone result in improved survival? By improving cachexia (i.e., leading to improved physical and physiological function) in cachexia, can patients become better able to tolerate anticancer therapies such as chemotherapy?

Composite endpoints which measure clinically relevant outcomes such as physical activity and quality of life are required in order to best assess the impact of interventions on cancer cachexia patients. Objective measures of function (as represented by physical activity) using advance ambulatory technology and integrated subjective quality of life parameters are likely to become standard practice in the clinical trial setting.

## Figures and Tables

**Table 1:** Diagnostic criteria for cachexia syndrome [6].

Weight loss of at least 5% in 12 months or less		
(or BMI <20 kg/m^2^)		
AND 3 of 5 From:	Decreased muscle strength	
	Fatigue	
	Anorexia	
	Low fat-free mass index	
	Abnormal biochemistry:	Increased inflammatory markers (CRP, IL-6)
		Anaemia (Hb < 12 g/dL)
		Low serum albumin (<3.2 g/dL)

Note: Fatigue is defined as physical and or mental weariness resulting from exertion; an inability to continue exercise at the same intensity with a resultant deterioration in performance.

Anorexia is defined as limited food intake (total caloric intake less than 20 kcal/kg body weight/day) or poor appetite.

Low-fat-free mass index represents lean tissue depletion (i.e., mid upper arm muscle circumference <10th percentile for age and gender' appendicle skeletal muscle index by DEXA <5.45  (kg/m^2^) in females and <7.25 in males).

**Table 2:** Modified Glasgow Prognostic Score (mGPS): an inflammation-based prognostic score [21].

Biochemical measure	Score
*C*-reactive protein ≤10 mg/L + Albumin ≥35 g/L	0
*C*-reactive protein ≤10 mg/L + Albumin <35 g/L	0
*C*-reactive protein >10 mg/L	1
*C*-reactive protein >10 mg/L + Albumin <35 g/L	2

**Table 3:** Endpoints for evaluating interventions in cancer cachexia.

Clinical	Functional	Biochemical
Nutritional status	Performance score (ECOG; Karnofsky)	Plasma fatty acid composition
Tolerance of diet	Quality of life scores	Pro-inflammatory cytokines
GI symptoms	Appetite	Acute phase protein reactants
Infections	Fatigue	
Survival	Physical activity as measured electronically [22]	
	Muscle strength	

**Table 4:** Pharmacological options for management of cachexia.

	Agent	Clinical effect (RCT)^#^	Hypothetical mechanism of action
Anabolic agents	Corticosteroids	Improves anorexia and weakness; no improvement in weight or calorie intake [23–25]; well tolerated; effects short lasting	Not established. May inhibit prostaglandin metabolism and central euphoric effect

	Nandrolone decanoate	Decrease in weight loss [26]	Not established. Promote protein nitrogen accumulation

	Oxandrolone	No published randomised clinical trials in cancer cohort	Not established

	Insulin	Increases whole body fat and carbohydrate intake [27]	Not established

	Adenosine Triphosphate (ATP)	Stabilises weight loss and increases energy intake[28]	Not established

Appetite stimulants	Progesterones: Megestrol acetate (MA) Medroxyprogesterone (MP)	Improves appetite, calorie intake and weight (not lean body mass) [29]	MA: may increase the central appetite stimulant neuropeptide YMP: reduces serotonin and cytokine production by PBMCs [30]

	Cannabinoids: Dronabinol	No benefit when added to MA; inferior to MA when used alone [31]. No increase in appetite or QoL [32]	May act on endorphin receptors, reduce prostaglandin synthesis or inhibit IL-1 secretion [33]

Cytokine inhibitors	Cyproheptadine	No improvement in weight gain [34]	Serotonin antagonist with antihistaminic properties

	Thalidomide	Attenuates weight loss, increases lean body mass [35]	Immunomodulatory: downregulates TNF-*α* (by destabilising mRNA [36]), NF*κ*B, pro-inflammatory cytokines, COX2 [37]

	Pentoxifylline	No improvement in appetite or weight in cachectic patients [38]	Phosphodiesterase inhibitor: inhibits TNF gene transcription

	Eicosapentaenoic acid (EPA)	Cochrane meta-analysis: insufficient evidence to establish whether EPA is better than placebo [39]	*In vitro *attenuates increased cAMP activity and lipolysis by LMF [40]

	Melatonin	Improves cachexia (term not defined) and one year survival increased in advanced NCSC lung cancer [41]	Immunomodulatory [42], Downregulates TNF production [43]

Anti-inflammatories	Non-steroid anti-inflammatory drugs	Reduced inflammatory markers, reduced resting energy expenditure, preservation of total body fat [44]	Not established. May downregulate systemic inflammatory response to tumour

^#^Results from randomised controlled trials (RCTs) are cited.
